# Multi‐marker DNA metabarcoding reveals spatial and sexual variation in the diet of a scarce woodland bird

**DOI:** 10.1002/ece3.10089

**Published:** 2023-05-17

**Authors:** Ewan H. Stenhouse, Paul Bellamy, Will Kirby, Ian P. Vaughan, Lorna E. Drake, Angela Marchbank, Trudy Workman, William O. C. Symondson, Pablo Orozco‐terWengel

**Affiliations:** ^1^ School of Biosciences Cardiff University Cardiff UK; ^2^ RSPB Centre for Conservation Science, The Lodge Sandy UK

**Keywords:** *Coccothraustes coccothraustes*, diet, Hawfinch, metabarcoding, omnivory

## Abstract

Avian diet can be affected by site‐specific variables, such as habitat, as well as intrinsic factors such as sex. This can lead to dietary niche separation, which reduces competition between individuals, as well as impacting how well avian species can adapt to environmental variation. Estimating dietary niche separation is challenging, due largely to difficulties in accurately identifying food taxa consumed. Consequently, there is limited knowledge of the diets of woodland bird species, many of which are undergoing serious population declines. Here, we show the effectiveness of multi‐marker fecal metabarcoding to provide in‐depth dietary analysis of a declining passerine in the UK, the Hawfinch (*Coccothraustes coccothraustes*). We collected fecal samples from (*n* = 262) UK Hawfinches prior to, and during, the breeding seasons in 2016–2019. We detected 49 and 90 plant and invertebrate taxa, respectively. We found Hawfinch diet varied spatially, as well as between sexes, indicating broad dietary plasticity and the ability of Hawfinches to utilize multiple resources within their foraging environments.

## Graphical abstract

Hawfinch (*Coccothraustes coccothraustes*) have been declining in the UK since the 1970s. Using DNA metabarcoding, we wanted to increase ecological knowledge associated with Hawfinch, to aid their recovery. We found 49 and 90 prey taxa in the diet, which can improve our knowledge of why Hawfinch persist within certain areas of the UK.

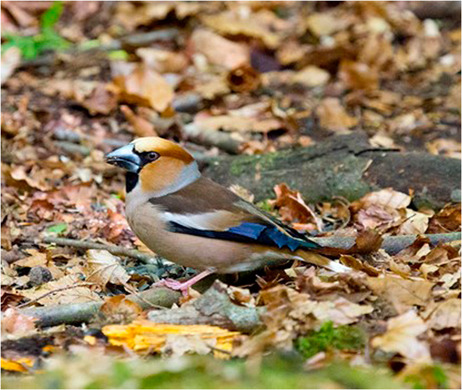

## 1 INTRODUCTION

Global biodiversity is currently undergoing a rapid decline, with many avian species experiencing significant population decreases (Spiller & Dettmers, 2019). It has been suggested that 13% of the world's avian species may experience extinction within 50 years, due to broad‐scale declines in both bird diversity and abundance recorded across avian groups (Alderson & Sander, 2022; Lindenmayer et al., 2018). Habitat specialists are deemed more vulnerable to population declines, as, while able to utilize resources more efficiently within their ecological niche, they are more vulnerable to habitat loss and degradation (Correll et al., 2019). Global patterns of species extinctions are underpinned by regional and local trends in populations (Inger et al., 2015), which are critical to determine species in need of conservation action (Pringle, 2017).

A vital aspect in understanding interactions within ecological communities is to accurately characterize species' diets, as this plays a pivotal role in defining ecological niches and determining individual fitness (Pompanon et al., 2012; Romano et al., 2020). Having an in‐depth understanding of birds' dietary niches allows an accurate understanding of the complex interactions which birds have within their environment (Hoenig et al., 2022). In turn, this provides essential information for the conservation and management of avian species and their associated habitats (O'Donnell et al., 2012; Ontiveros et al., 2005). Intraspecific dietary niche separation is important for ecological dynamics (Cloyed & Eason, 2017) and has a number of drivers. Environmental factors such as habitat type and quality may influence diet due to potential changes in species composition between habitat types (β‐diversity; Shutt et al., 2019). Within forests, it has been found that invertebrate species richness can differ between tree taxa (Murakami et al., 2008; Shutt et al., 2019). Intrinsic factors, such as sex can also be a source of dietary variation, driven by morphological and behavioral differences, as well as differing nutritional requirements (da Silva et al., 2020; Mata et al., 2016). Environmental factors can also interact with intrinsic factors to influence dietary variation between demographic groups, such as dietary variation between sexes only being apparent during the breeding season, when males and females have differing reproductive demands (da Silva et al., 2020; Durell et al., 1993).

The Hawfinch (*Coccothraustes coccothraustes*) is one of many woodland specialists to have shown major declines in the UK (Kirby et al., 2018). Hawfinch breed across the Palearctic, with Britain its western range limit (Kirby et al., 2015). Hawfinch ecology is poorly understood, and in Britain, Hawfinch are now too rare to have regular status assessments by national annual monitoring schemes (Kirby et al., 2015). Instead, population change is inferred from distribution data compiled from bird atlas surveys (Balmer et al., 2013). These atlas data indicate a 76% reduction in the number of 10 km squares occupied between 1968 and 2011 (Kirby et al., 2015, 2018). This decline was previously evidenced by Langston et al. (2002), who estimated a 40% population decline between the mid‐1980s and late‐1990s. Hawfinch show a very localized distribution within the UK, with population strongholds exhibiting a strong westerly bias (Kirby et al., 2018), and the factors causing their decline are unknown. Landscape modification, decreased invertebrate abundance, and changes in woodland management have been suggested (Fuller et al., 2005; Kirby et al., 2018). Further potential contributory factors may include under‐planting of ancient woodland with conifers in the 1970s, and a storm in 1987 which caused the loss of many cherry trees, which are thought to be an important food resource for Hawfinch (Kirby et al., 2018; Spencer & Kirby, 1992).

Hawfinch dietary studies are limited, with all previous information obtained through visual observation (Mountford, 1957; Newton, 1967). Hawfinch are thought to be dietary specialists adapted to utilize large seeded tree species due to their large and powerful beak (Mountford, 1957). During the breeding season (typically from April to June), Hawfinch have been observed to feed on buds and flowers of cherry (*Prunus* sp.), hornbeam (*Carpinus betulus*), beech (*Fagus sylvatica*), and Wych elm (*Ulmus glabra*; Mountford, 1957). Hawfinch have also been observed to incorporate invertebrates into their diet during the breeding season, consuming Lepidoptera, Coleoptera, Hemiptera, Annelida, Gastropoda, and Araneae (Mountford, 1957).

DNA metabarcoding is frequently utilized to assess the diet of a range of organisms (Cuff et al., 2021; Davies et al., 2022; Evens et al., 2020; Fernandes et al., 2023; Forsman et al., 2022; Kartzinel & Pringle, 2020; Thompson & Newmaster, 2014; Zalewski et al., 2021). Metabarcoding requires minimal a priori knowledge of the target organism's diet (Alberdi et al., 2017; Valentini et al., 2009), and a wide range of ingested taxa can be identified to fine taxonomic levels (Hoenig et al., 2022). Morphology‐based methods often record ingested taxa at a coarse taxonomic resolution, missing subtle differences in the taxa consumed (Alberdi et al., 2017). This limits the opportunities to make fine scale inferences relating to species' ecology (da Silva et al., 2020; Mata et al., 2016). The accurate identification of components within an omnivorous diet is, however, still considered challenging (da Silva et al., 2019; De Barba et al., 2014; Tercel et al., 2021). Due to the costly, laborious, and taxonomically demanding nature of exploring omnivorous diet, studies attempting to elucidate all dietary aspects are rare (Pompanon et al., 2012; Robeson et al., 2018; Tercel et al., 2022).

Metabarcoding studies have been used to successfully identify details of the diet of farmland birds (Cabodevilla et al., 2021) and insectivorous species (Evens et al., 2020; McClenaghan et al., 2019; Mitchell et al., 2021). Metabarcoding therefore has the potential to improve our knowledge of the diet of omnivorous woodland passerines. Despite this, studies focusing on omnivorous birds are rare (but see da Silva et al., 2020; Spence et al., 2022; Tang et al., 2022). There are currently no studies which have used metabarcoding to reconstruct the diet of UK Hawfinch populations. Incomplete information on dietary breadth and key dietary resources hampers the identification of conservation strategies and delays the preservation of key food resources attributing to Hawfinch persistence. By exploring dietary variation between populations and demographic groups, conservation efforts can be better designed to ensure the survival of Hawfinches. Here, we undertook the first extensive metabarcoding dietary analysis of Hawfinch in the UK to obtain in‐depth dietary information across spatial scales and demographic groups. Combining broad‐coverage plant and invertebrate DNA metabarcoding primers, we aimed to (i) document the dietary composition of UK Hawfinches; (ii) test whether diet varies spatially, and (iii) test whether diet differs between males and females.

## 2 MATERIALS AND METHODS

### 2.1 Study sites and field data collection

**FIGURE 1:**
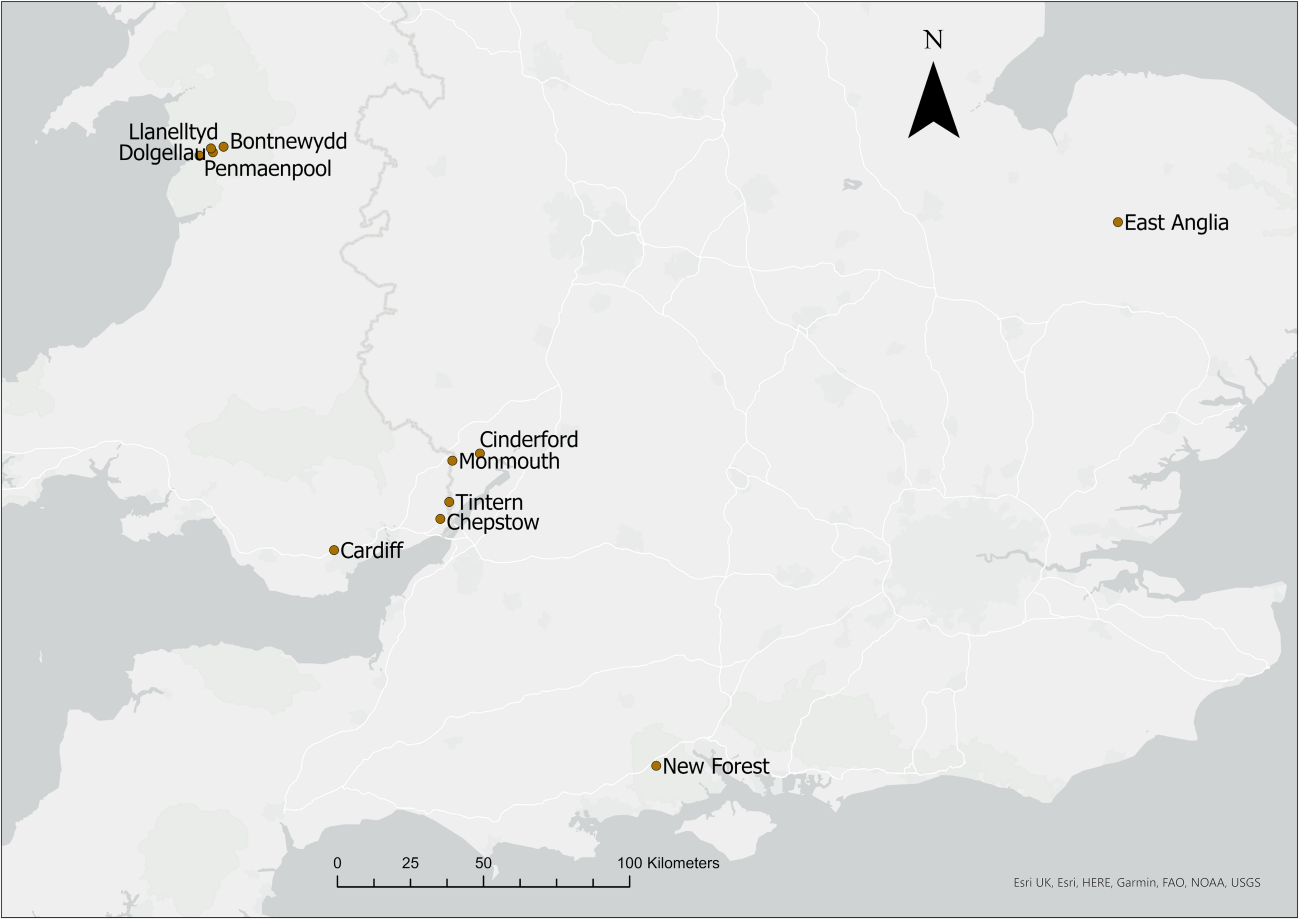
Locations of study sites where fecal samples were collected are shown as red dots. Map was produced on ArcGis v3.16.8 (QGIS development team, 2021).

Fieldwork was conducted in the period March to July of 2016–2019 at 11 woodland feed sites in the UK. Sites selected were pre‐existing Hawfinch ringing study areas within the Wye Valley (Cinderford, Chepstow, Monmouth, and Tintern), north Wales (Bontnewydd, Dolgellau, Llanellytd and Penmaenpool), north Cardiff, the New Forest, and East Anglia (Figure 1). Sampling sizes per region are displayed in Table 1. The artificial feed sites used to attract Hawfinches for capture have been operational for a number of years within regions of Hawfinch population strongholds (Clements, 2013; Kirby et al., 2018). All feed sites were baited with supplementary sunflower (*Helianthus* sp.) seeds, provided ad libitum from December to July. Study sites were broadly typical of British mixed broadleaved woodland, with sites in the Wye Valley and north Wales dominated by beech, oak, and ash (*Fraxinus excelsior*). The study site located in East Anglia was a mixed woodland consisting of lime (*Tilia* sp.), ash, and maples (*Acer* sp.). The New Forest site was dominated by oak, with an understorey flora comprising of Holly (*Ilex* sp.) and bramble (*Rubus* sp.). All site locations are approximate for anonymity.

**TABLE 1:** The sampling effort of Hawfinch captured across regions of the UK, broken down by sex, age, and year.

Region	Number of Hawfinch sampled (total)	Sex	Age	Year
North Wales	115	Male = 60 Female = 55	Adults = 94 Juveniles = 21	2016 = 15
				2017 = 34
				2018 = 9
				2019 = 57
North Cardiff	7	Male = 4 Female = 3	Adults = 7 Juveniles = 0	2016 = 0
				2017 = 0
				2018 = 2
				2019 = 5
New Forest	19	Male = 14 Female = 5	Adults = 17 Juveniles = 2	2016 = 0
				2017 = 0
				2018 = 3
				2019 = 16
East Anglia	7	Male = 3 Female = 4	Adults = 6 Juveniles = 1	2016 = 0
				2017 = 0
				2018 = 0
				2019 = 7
Wye Valley	138	Male = 78 Female = 60	Adults = 134 Juveniles = 0	2016 = 1
				2017 = 0
				2018 = 65
				2019 = 72

Hawfinches were caught by trained bird ringers, operating under British Trust for Ornithology (BTO) approved licenses using either mist or whoosh nets. Sex was determined through secondary wing feather coloration following Svensson (1992). All birds were aged as adults or juveniles, with birds aged through width of the outer web of the sixth primary feather and sharpness of the greater alula feather, following Fornasari et al. (1994). Juveniles were defined as young of that year which had already fledged. Hawfinch show strong sexual plumage color dimorphism of secondary feathers, enabling the sexing of individuals in both the non‐breeding and breeding season (Svensson, 1992). We placed Hawfinches within individual clean, paper bags which were then placed inside a cotton bag and left for 10–20 min until the bird defecated. To avoid excessive stress, if birds had not defecated within this time frame they were processed and released. We removed each fecal sample using a new, clean, plastic toothpick to minimize contamination. Samples were frozen to −20°C after collection.

### 2.2 Dietary analysis

We undertook DNA extractions in a dedicated pre‐PCR laboratory. We extracted DNA from a total of 365 fecal samples using the Qiagen QIAamp DNA Stool Mini Kit (Qiagen) with modifications designed to improve DNA yield from avian feces, following Shutt et al. (2020). We extracted samples in batches of 23 with an additional negative control containing no DNA. To characterize Hawfinch diet, we used universal primers UniplantF, 5′‐TGTGAATTGCARRATYCMG‐3′ and UniPlantR 5′‐CCCGHYTGAYYTGRGGTCDC‐3′ to amplify a 187–387‐bp fragment covering the ITS2 region of plant nuclear DNA (Moorhouse‐Gann et al., 2018; Tercel et al., 2022). For amplification of invertebrate DNA, we used the universal primers mlCOIintF, 5′‐GGWACWGGWTGAACWGTWTAYCCYCC‐3′ (Leray et al., 2013) and Nancy 5′‐ACTAGCAGTACCCGGTAAAATTAAAATATAAACTTC‐3′, (Simon et al., 1992), following selection and modification by Stockdale (2018) for amplification of a 306‐bp fragment of the COI region (Davies et al., 2022). Stockdale (2018) validated primer sets to ensure DNA amplification of the expected range of taxa. The PCR process involved amplification of the target regions with the addition of a unique combination of 10‐bp molecular identifier tags (MID‐tags), with samples having a unique pairing of forward and reverse tags for subsequent sample identification. This was undertaken separately for each marker, so all samples could be uniquely identified for both markers. Within each PCR 96‐well plate, 12 negative (extraction and PCR) and two positive controls were included following Taberlet et al. (2018). We categorized all products from each individual PCR plate based on band brightness after gel electrophoresis (very faint, faint, medium, bright). We quantified the DNA concentration from a minimum of three representative PCR products per plate from each brightness category using a high sensitivity assay with a Qubit Flourometer (Thermo Fisher Scientific) to confirm whether estimating relative DNA concentration by eye from a gel photo was accurate. For each marker, we pooled each PCR plate according to concentrations determined by the Qubit Fluorometer to ensure equimolar concentration of all PCR products in each pool.

We multiplexed the amplicons into five pools, each containing between 63 and 186 samples. We undertook library preparation for Illumina sequencing using a NEXTflex Rapid DNA‐Seq kit (Bioo Scientific), with a unique adapter added to each pool for subsequent bioinformatic identification. We diluted pools to 4 nM and quantified them using Qubit dsDNA High‐sensitivity Assay Kits. Finally, we combined the diluted pools equimolarly and sequenced the pools on a MiSeq desktop sequencer via a v2 chip with 2 × 250 bp paired end reads (expected capacity 24–30,000,000 reads). Due to the unbalanced nature of the amplicon libraries, we added a 15% PhiX buffer to the sequencing run to improve crosstalk and phasing calculations.

### 2.3 Identification of dietary taxa

The Illumina runs generated 6,328,388 and 12,307,560 ITS2 and COI reads, respectively. The bioinformatics pipeline was undertaken following Drake et al. (2021) and Davies et al. (2022). We used FastP (Chen et al., 2018) to trim, align, and quality check sequencing reads. Tagged reads were labeled by unique sample ID using Mothur (Schloss et al., 2009) and subsequently demultiplexed. We implemented Unoise 3 within Usearch (Edgar, 2016) to remove chimeras and cluster reads to generate zero‐radius Operational Taxonomic Units (zOTUs) using a 100% clustering threshold, as well as creating a read abundance matrix for zOTUs produced. The Blastn algorithm in Blast+ (Camacho et al., 2009) matched reference sequences contained within GenBank to the sequences generated. We used Megan6 (Huson et al., 2016) to identify unique dietary items using the top hit for each zOTU (based on bit‐score). As described in Drake et al. (2021), a minimum percentage match of 97% was deemed suitable for species or genus‐level classification. Genus‐level classification was only assigned if multiple zOTUs matched with several species of the same genus. A minimum percentage match of 95% was implemented for classification to the family level. All read counts less than the maximum in unused‐MID tag combinations and negative controls for each respective zOTU were removed prior to statistical analysis. For example, if a negative control had a read count of 150 sequences for a known dietary zOTU, we removed 150 reads from all samples the zOTU was found in. We opted not to use a standard threshold for retaining zOTUs (such as ten reads per sample, as commonly used within dietary metabarcoding studies). Instead, we determined thresholds for read removal using the percentage at which known artifacts assigned to known positive controls were removed (Cuff et al., 2021; Davies et al., 2022; Drake et al., 2021). To be retained, the read count of a zOTU had to be greater than 3% and 1% of the maximum read count for all detections of that zOTU across all samples for ITS2 and COI, respectively (Davies et al., 2022; Appendices [Link], [Link], [Link], [Link]). This further cleaning step was performed to account for over‐represented taxa tag jumping or “leaking” from samples with extremely high read counts across multiple samples. Data from respective ITS2 and COI libraries were aggregated together to form a single taxon list for each marker, and non‐dietary species such as fungi and gastrotrichs were removed.

### 2.4 Statistical analysis

For all statistical analyses, the presence/absence of each taxonomic unit within a sample was used instead of read count. The use of relative read abundance (RRA) was not deemed suitable, due to the large number of reads detected from the high densities of sunflower seeds provided at all sites, which may skew ecological conclusions generated from RRA data. Furthermore, there are inherent biases present throughout the HTS workflow, including differential DNA extraction success and PCR amplification rates between taxa detected within the diet (Lamb et al., 2019). Additionally, count‐based inferences are not advised if little a priori knowledge of the communities analyzed exists (Lamb et al., 2019). Control samples were excluded from the analyses. All statistical analyses were carried out in R version 4.2.2 (R Core Team, 2020) unless otherwise stated. All analysis was undertaken at the genus level to standardize taxonomic resolution, as not all taxa could be identified to species.

To evaluate the most prevalent plant and invertebrate taxa within Hawfinch diet, we calculated frequency of occurrence (FOO). We did this by totalling the number of instances that a given taxon occurred across all hawfinch samples. We then calculated this as a percentage of the total number of samples (% FOO), by dividing the frequency of occurrence by the total number of hawfinch fecal samples collected and multiplying by 100. We also aimed to quantify dietary diversity and reveal if the number of samples collected in this study was sufficient to represent Hawfinch diet within the UK. To achieve this, we used coverage‐based rarefaction and extrapolation. We used Hill diversity metrics to estimate species diversity (Roswell et al., 2021; Tercel et al., 2022) as opposed to species‐accumulation curves, as these have been shown to poorly represent true community diversity (Roswell et al., 2021). Coverage, as defined by Roswell et al. (2021) “describes to what extent a sample captures the true diversity of the whole community, including species that have not yet been detected”. For example, a coverage of 0.80 means that 20% of the individuals in the community being sampled belong to species that have not been found (Roswell et al., 2021). We produced coverage‐based rarefaction and extrapolation curves and estimates of species richness (Hill‐richness), Shannon's entropy (Hill‐Shannon), and Simpson's index (Hill‐Simpson) in R using the *iNEXT* package (Hsieh et al., 2016). For all plots, 95% confidence intervals around estimates are shown (Roswell et al., 2021; Stillman et al., 2022; Tercel et al., 2022).

To test whether Hawfinch diet differed between populations and sexes, we applied a single multivariate generalized linear model (MGLM) containing the predictor variables region and sex, using the function *manyglm* within the package *mvabund* (Wang et al., 2012). Due to the small numbers of juveniles (Table 1), they were removed from all analyses. We broadly categorized regions into the following: Wye Valley, north Wales, north Cardiff, New Forest, and East Anglia, as some regions contained multiple catching sites. If an individual was sampled more than once, data was used from the first capture only to avoid pseudo‐replication. Year of sampling was included as a covariate in all models to control for any variation through time. To assess if any specific plant or invertebrate taxa were responsible for dietary differences regionally or between sexes, *p*‐values from univariate tests were extracted with the function *anova*.*manyglm* using the *p*.*uni = “adjusted”* argument. This adjusts *p*‐values using Holm's step down resampling algorithm (Westfall & Young, 1993). We applied parametric bootstrap (Monte Carlo) resampling, ensuring inferences took into account correlation between variables, as this is recommended for presence‐absence multivariate data (Wang et al., 2012). When necessary, we performed pairwise comparisons using the *pairwise*. *comp* function of *anova*.*manyglm*. For all models, we checked quantile‐quantile (Q–Q) diagnostic plots for normality, with homoscedasticity checked by plotting Dunn‐Smyth residuals against fitted linear predicted values (Bates et al., 2015; Wang et al., 2012). We visualized dietary differences using non‐metric multidimensional scaling analysis (NMDS) via the function *metaMDS* in the *vegan* package (Oksanen & Wagner, 2019). The nMDS was performed with Jaccard dissimilarities in two‐dimensional space (*k* = 2). We produced spider plots using nMDS results via *ordispider* and plotted through ggplot2 (Wickham, 2016). Singletons and outliers were removed to visually improve the ordination.

## 3 RESULTS

### 3.1 Overview of Hawfinch diet composition

**FIGURE 2:**
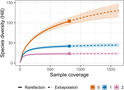
Species diversity by number of dietary detections found in Hawfinch fecal samples. Line colors determine the different diversity estimates: Species richness (Hill‐richness) = 0, orange line with terminal square; Shannon diversity (Hill‐Shannon) = 1, blue line with terminal circle; Simpson diversity (Hill‐Simpson) = 2, purple line with terminal triangle. Solid lines = observed, dashed lines = extrapolated. Confidence intervals (95%) are denoted by shading around the line.

**FIGURE 3:**
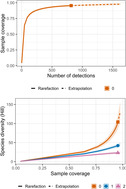
The level of community coverage provided in Hawfinch fecal samples. Coverage is defined by Roswell et al. (2021) as “describes to what extent a sample captures the true diversity of the whole community, including species that have not yet been detected”. Line colors determine the differing diversity estimates: Species richness (Hill‐richness) = 0, orange line with terminal square; Shannon diversity (Hill‐Shannon) = 1, blue line with terminal circle; Simpson diversity (Hill‐Simpson) = 2, purple line with terminal triangle. Solid lines = observed, dashed lines = extrapolated. Confidence intervals (95%) are denoted by shading around the line. Left: sample coverage by number of dietary detections. Right: species diversity by sample coverage.

Across the 262 Hawfinch samples, there were 815 detections from a total of 139 plant and invertebrate taxa. The species richness (Hill‐richness) metric showed the highest dietary diversity estimate when compared with Shannon and Simpson diversity metrics (Figure 2). We calculated that 95.5% (±95% CI: 17.6%) of possible dietary items were detected, indicating a high degree of sampling completeness (Figure 3). These diversity estimates indicate Hawfinch consume numerous rarely eaten individual taxa, as opposed to focusing on a few commonly eaten, or evenly consuming dietary taxa, as Hill‐richness is most sensitive to rare species (Roswell et al., 2021; Tercel et al., 2022).

We successfully amplified plant DNA from 262 fecal samples. We identified 49 plant zOTUs belonging to 17 families, of which 86% could be identified to species and 100% to genus (Table 2). The three most frequently consumed taxa were beech (38.5% of samples), sunflower seed (*Helianthus* sp.), provided ad libitum throughout the year at all feed sites (30.5% of samples), and hornbeam (16.0% of samples). English oak (*Quercus robur*) and wild cherry (*Prunus avium*) were also frequently detected (14.9% and 11.5%, respectively). While tree species dominated Hawfinch diet, we detected herbaceous plant taxa such as dandelions (*Taraxacum* sp.) and *Rubus* sp. (0.4% and 1.5%, respectively), which were not known to be consumed by Hawfinch.

**TABLE 2:** The percentage of Hawfinch fecal samples testing positive for plant dietary items broken down by sampling region.

Percentage of samples testing positive for plant taxa (%FOO)						
Taxon	All (*n* = 262)	North Wales (*n* = 113)	Wye Valley (*n* = 116)	New Forest (*n* = 19)	North Cardiff (*n* = 7)	East Anglia (*n* = 7)
*Acer campestre*	0.4	0	0.9	0	0	0
*Acer japonicum*	0.4	0.9	0	0	0	0
*Acer platanoides*	0.4	0.9	0	0	0	0
*Acer pseudoplatanus*	4.2	4.4	0.9	0	14.3	57.1
*Alnus glutinosa*	1.5	0	0.9	15.8	0	0
*Anthoxanthum odoratum*	0.4	0.9	0	0	0	0
*Anthoxanthum ovatum*	0.4	0.9	0	0	0	0
*Betula pendula*	5	7.1	0.9	21.1	0	0
*Betula pubescens*	6.5	10.6	0	26.3	0	0
*Brassica oleracea*	0.8	0.9	0.9	0	0	0
*Brassica* sp.	0.4	0.9	0	0	0	0
*Carpinus betulus*	16	20.4	8.6	0	28.6	100
*Carpinus laxiflora*	0.8	1.8	0	0	0	0
*Carpinus* sp.	0.4	0.9	0	0	0	0
*Corylus avellana*	0.4	0.9	0	0	0	0
*Dactylis glomerata*	0.4	0.9	0	0	0	0
*Fagus sylvatica*	38.5	17.7	62.1	26.3	57.1	0
*Fraxinus excelsior*	2.7	2.7	0.9	10.5	14.3	0
*Hedera helix*	1.5	0	3.4	0	0	0
*Helianthus* sp.	30.5	50.4	11.2	21.1	14.3	71.4
*Holcus lanatus*	1.1	0.9	0.9	5.3	0	0
*Ilex aquifolium*	5.3	3.5	1.7	42.1	0	0
*Larix decidua*	1.1	0.9	0.9	0	14.3	0
*Nothofagus obliqua*	1.5	1.8	0.9	5.3	0	0
*Poa infirma*	0.4	0	0.9	0	0	0
*Poa trivialis*	0.8	1.8	0	0	0	0
*Prunus avium*	11.5	13.3	12.9	0	0	0
*Prunus cerasifera*	0.4	0.9	0	0	0	0
*Prunus domestica*	0.8	0.9	0	5.3	0	0
*Prunus spinosa*	0.4	0	0	5.3	0	0
*Quercus cerris*	0.4	0.9	0	0	0	0
*Quercus hartwissiana*	2.3	0.9	2.6	10.5	0	0
*Quercus petraea*	9.9	4.4	12.1	31.6	0	14.3
*Quercus robur*	14.9	9.7	12.9	57.9	0	28.6
*Quercus* sp.	6.5	5.3	6	15.8	0	14.3
*Ranunculus repens*	0.4	0	0.9	0	0	0
*Rosa arvensis*	0.4	0	0.9	0	0	0
*Rosa glauca*	0.4	0	0.9	0	0	0
*Rosa moschata*	0.4	0.9	0	0	0	0
*Rubus* sp.	1.5	0.9	1.7	5.3	0	0
*Rubus vestitus*	0.4	0	0.9	0	0	0
*Salix* sp.	1.5	2.7	0.9	0	0	0
*Sorbus aucuparia*	1.5	2.7	0.9	0	0	0
*Taraxacum officinale*	0.4	0	0.9	0	0	0
*Taxus baccata*	2.3	0.9	3.4	5.3	0	0
*Ulmus glabra*	8	0	16.4	0	28.6	0
*Urtica* sp.	0.4	0	0.9	0	0	0
*Veronica chamaedrys*	0.4	0.9	0	0	0	0
*Viola* sp.	0.4	0.9	0	0	0	0

*Note*: Frequency of occurrence (%FOO) is expressed as a percentage.

The same 262 samples were also tested for invertebrate DNA. We successfully amplified invertebrate DNA from 120 fecal samples. 90 invertebrate zOTUs, belonging to nine orders were identified, with 92% identified to species level and 100% to genus (Table 3). Lepidoptera dominated Hawfinch diet (58%), followed by Diptera (11%) and Hymenoptera (7%). The most common taxa detected were the winter moth (*Operophtera brumata*; 26.7% of samples), mottled umber (*Erannis defoliaria*; 22.5% of samples), and tree slug (*Lehmannia marginata*; 22.5% of samples). Chestnut moth (*Conistra vaccinii*) and the Satellite moth (*Eupsilia transversa*) were also frequently consumed (15% and 14.2%, respectively).

**TABLE 3:** The percentage of Hawfinch fecal samples testing positive for invertebrate dietary items broken down by sampling region.

Percentage of samples testing positive for invertebrate taxa (%FOO)							
Taxon	Order	All (*n* = 120)	North Wales (*n* = 43)	Wye Valley (*n* = 56)	New Forest (*n* = 13)	North Cardiff (*n* = 5)	East Anglia (*n* = 3)
*Acleris rhombana*	Lepidoptera	0.8	0	1.8	0	0	0
*Adela reaumurella*	Lepidoptera	0.8	0	0	8.3	0	0
*Aethalura punctulata*	Lepidoptera	1.7	2.3	0	8.3	0	0
*Agelastica alni*	Coleoptera	2.5	0	3.6	8.3	0	0
*Agriopis aurantiaria*	Lepidoptera	0.8	2.3	0	0	0	0
*Agriopis marginaria*	Lepidoptera	2.5	4.7	0	8.3	0	0
*Agrochola circellaris*	Lepidoptera	2.5	0	3.6	0	20	0
*Agrochola macilenta*	Lepidoptera	1.7	2.3	1.8	0	0	0
*Aleimma loeflingiana*	Lepidoptera	4.2	0	7.1	8.3	0	0
*Amphipyra berbera*	Lepidoptera	13.3	0	12.5	58.3	40	0
*Amphipyra pyramidea*	Lepidoptera	5.8	0	7.1	8.3	20	33.3
*Andricus curvator*	Hymenoptera	1.7	0	1.8	8.3	0	0
*Anorthoa munda*	Lepidoptera	2.5	4.7	1.8	0	0	0
*Anyphaena accentuata*	Araneae	5.8	16.3	0	0	0	0
*Apocheima pilosaria*	Lepidoptera	0.8	2.3	0	0	0	0
*Apotomis turbidana*	Lepidoptera	0.8	2.3	0	0	0	0
*Araneus triguttatus*	Araneae	1.7	0	1.8	8.3	0	0
*Archips crataeganus*	Lepidoptera	0.8	0	0	0	0	33.3
*Archips xylosteana*	Lepidoptera	2.5	0	0	25	0	0
*Biorhiza pallida*	Lepidoptera	0.8	0	0	8.3	0	0
*Blastobasis adustella*	Lepidoptera	0.8	0	1.8	0	0	0
*Charmon* sp.	Hymenoptera	0.8	2.3	0	0	0	0
*Cimbex femoratus*	Hymenoptera	4.2	9.3	0	8.3	0	0
*Cimbex* sp.	Hymenoptera	0.8	2.3	0	0	0	0
*Clausilia bidentata*	Lepidoptera	0.8	0	1.8	0	0	0
*Clubiona brevipes*	Pulmonata	0.8	0	0	8.3	0	0
*Coleophora flavipennella*	Lepidoptera	1.7	2.3	0	8.3	0	0
*Colotois pennaria*	Lepidoptera	6.7	4.7	10.7	0	0	0
*Conistra* sp.	Lepidoptera	0.8	2.3	0	0	0	0
*Conistra vaccinii*	Lepidoptera	15	14	14.3	33.3	0	0
*Cosmia trapezina*	Lepidoptera	5.8	0	5.4	33.3	0	0
*Craniophora ligustri*	Lepidoptera	0.8	2.3	0	0	0	0
*Culicoides impunctatus*	Diptera	1.7	4.7	0	0	0	0
*Culicoides obsoletus*	Diptera	0.8	0	1.8	0	0	0
*Cyzenis albicans*	Diptera	0.8	2.3	0	0	0	0
*Drepana falcataria*	Lepidoptera	1.7	2.3	0	8.3	0	0
*Ectropis crepuscularia*	Lepidoptera	2.5	4.7	1.8	0	0	0
*Egle groenlandica*	Diptera	0.8	2.3	0	0	0	0
*Empoasca decipiens*	Hemiptera	0.8	0	0	8.3	0	0
*Epinotia immundana*	Lepidoptera	0.8	0	0	8.3	0	0
*Epirrita autumnata*	Lepidoptera	5	14	0	0	0	0
*Epirrita christyi*	Lepidoptera	7.5	14	5.4	0	0	0
*Epirrita dilutata*	Lepidoptera	4.2	2.3	7.1	0	0	0
*Epuraea melanocephala*	Coleoptera	0.8	2.3	0	0	0	0
*Erannis defoliaria*	Lepidoptera	22.5	48.8	10.7	0	0	0
*Eriocrania* sp.	Lepidoptera	2.5	7	0	0	0	0
*Eudemis profundana*	Lepidoptera	5.8	0	3.6	33.3	0	33.3
*Eupithecia abbreviata*	Lepidoptera	2.5	7	0	0	0	0
*Eupsilia transversa*	Lepidoptera	14.2	0	26.8	8.3	0	33.3
*Formica pratensis*	Hymenoptera	0.8	0	0	8.3	0	0
*Gypsonoma dealbana*	Lepidoptera	3.3	0	3.6	16.7	0	0
*Hemerobius micans*	Neuroptera	1.7	2.3	1.8	0	0	0
*Hydriomena furcata*	Lepidoptera	1.7	0	3.6	0	0	0
*Hyposoter* sp.	Hymenoptera	0.8	0	0	8.3	0	0
*Lehmannia marginata*	Pulmonata	22.5	27.9	25	0	20	0
*Limax cinereoniger*	Pulmonata	0.8	2.3	0	0	0	0
*Neomyia cornicina*	Diptera	0.8	2.3	0	0	0	0
*Neriene peltata*	Araneae	0.8	0	1.8	0	0	0
*Neuroterus quercusbaccarum*	Hymenoptera	10	9.3	5.4	41.7	0	0
*Oncopsis speciosa*	Hemiptera	0.8	2.3	0	0	0	0
*Operophtera brumata*	Lepidoptera	26.7	16.3	28.6	33.3	40	100
*Operophtera fagata*	Lepidoptera	4.2	11.6	0	0	0	0
*Orthosia cerasi*	Lepidoptera	11.7	11.6	10.7	16.7	0	33.3
*Orthosia cruda*	Lepidoptera	3.3	0	3.6	16.7	0	0
*Orthosia incerta*	Lepidoptera	2.5	2.3	0	16.7	0	0
*Pandemis cerasana*	Lepidoptera	4.2	0	5.4	16.7	0	0
*Pandemis cinnamomeana*	Lepidoptera	0.8	0	1.8	0	0	0
*Philodromus aureolus*	Araneae	0.8	0	0	8.3	0	0
*Philodromus* sp.	Araneae	1.7	0	3.6	0	0	0
*Phycita roborella*	Lepidoptera	1.7	0	1.8	8.3	0	0
*Phyllobius maculicornis*	Coleoptera	0.8	0	0	0	0	33.3
*Polydrusus undatus*	Coleoptera	0.8	0	1.8	0	0	0
*Psoricoptera gibbosella*	Lepidoptera	0.8	0	0	8.3	0	0
*Ptycholoma lecheana*	Lepidoptera	7.5	4.7	12.5	0	0	0
*Quercusia quercus*	Lepidoptera	2.5	2.3	1.8	8.3	0	0
*Rhynchaenus fagi*	Coleoptera	4.2	0	1.8	16.7	40	0
*Satyrium* sp.	Lepidoptera	2.5	0	3.6	0	20	0
*Scathophaga stercoraria*	Diptera	0.8	2.3	0	0	0	0
*Selenia tetralunaria*	Lepidoptera	0.8	2.3	0	0	0	0
*Stomoxys calcitrans*	Diptera	0.8	2.3	0	0	0	0
*Syrphus ribesii*	Diptera	1.7	0	0	0	0	66.7
*Syrphus torvus*	Diptera	1.7	0	0	0	0	66.7
*Taeniothrips inconsequens*	Thysanoptera	12.5	4.7	17.9	0	60	0
*Tetragnatha obtusa*	Araneae	0.8	2.3	0	0	0	0
*Thrips major*	Thysanoptera	1.7	0	3.6	0	0	0
*Thrips minutissimus*	Thysanoptera	7.5	7	10.7	0	0	0
*Tipula paludosa*	Diptera	0.8	0	1.8	0	0	0
*Tortricodes alternella*	Lepidoptera	12.5	0	19.6	25	20	0
*Vitrina pellucida*	Pulmonata	0.8	2.3	0	0	0	0
*Ypsolopha alpella*	Lepidoptera	1.7	0	0	16.7	0	0

*Note*: Frequency of occurrence (%FOO) is expressed as a percentage.

### 3.2 Variation in diet composition between sites and sex

**FIGURE 4:**
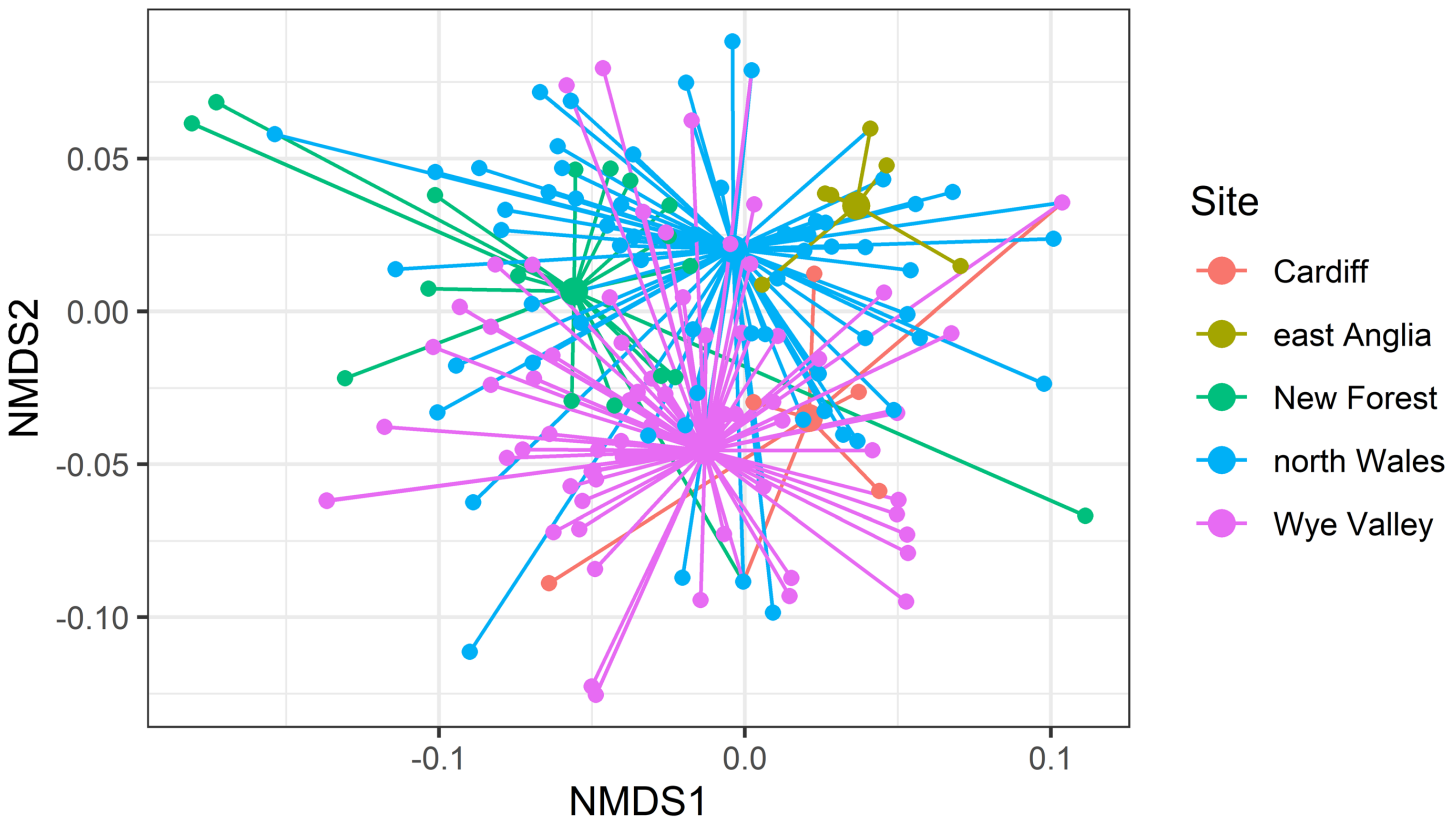
Spider plot for plant taxa consumed by Hawfinch in different geographic regions. Smaller nodes represent individual Hawfinch with connecting lines joining the individual to the mean centroid (larger nodes) of its region. Stress = 0.06.

MGLMs revealed that diet differed significantly between sampling regions (MGLM: Wald = 416.5, *p* = <.001; Figure 4). Pairwise comparisons revealed significant differences between all pairs of sampling locations except the Wye Valley vs north Cardiff (Wald = 11.8, *p* = .32; Appendix S5). Six genera showed a significant GLM result: *Betula* sp. (site: Wald = 21.3, *p* = .002), *Carpinus* sp. (site: Wald = 36.0, *p* = .001), *Fagus* sp. (site: Wald = 56.4, *p* = .001), *Helianthus* sp. (site: Wald = 51.6, *p* = .001), *Quercus* sp. (site: Wald = 19.2, *p* = .002), and *Ulmus* sp. (site: Wald = 30.5, *p* = .001; Appendix S5). Specifically, *Fagus* sp. and *Ulmus* sp. were detected with the highest frequency in the Wye Valley (62.1% and 16.4%, respectively), with birds sampled from north Wales showing the highest frequency for *Helianthus* sp. (50.9%). *Quercus* sp. were detected at the highest frequency in Hawfinches sampled within the New Forest (57.9%). Diet varied between years (Wald 396.3, *p* = .001), with four genera showing a significant GLM result: *Betula* sp. (year: Wald = 24.7, *p* = .002), *Carpinus* sp. (year: Wald = 20.7, *p* = .001), *Fagus* sp. (year: Wald = 18.5, *p* = .001), and *Helianthus* sp. (year: Wald = 16.6, *p* = .001).

**FIGURE 5:**
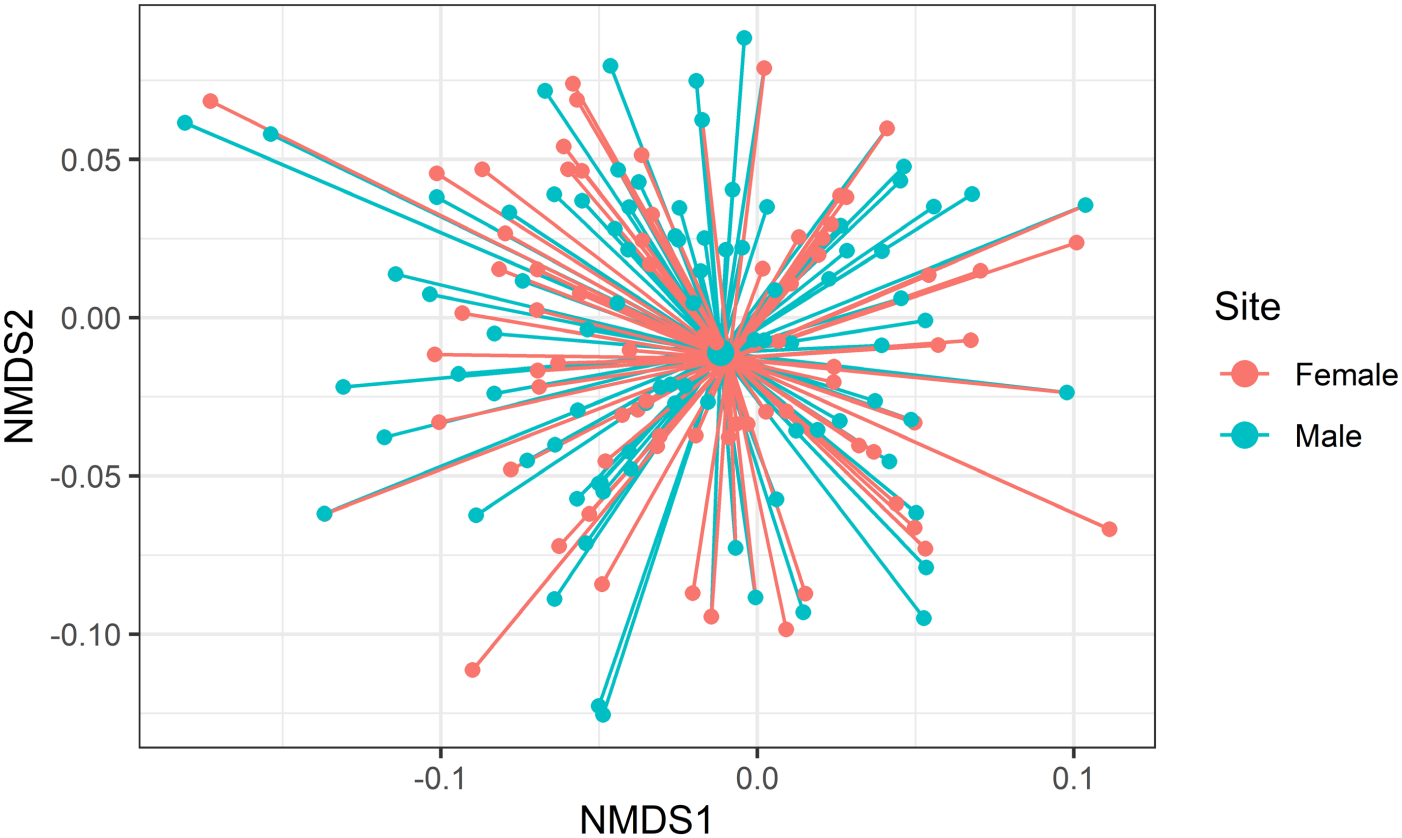
Spider plot for invertebrate taxa consumed by male and female Hawfinch. Smaller nodes represent individual Hawfinch with connecting lines joining the individual to the mean centroid (larger nodes) of its sex. Stress = 0.06.

Dietary composition differed significantly between sexes (MGLM: Wald = 148.4, *p =* <.001; Figure 5); however, no univariate tests showed significant differences (Appendix S5). Many taxa were consumed at different frequencies between sexes. Females were found to be feeding more frequently on invertebrates than males (52% of all invertebrate taxa detected were consumed more frequently by females) however, differences for all taxa were smaller than 10% (Table 4). Artificially provided sunflower seed (*Helianthus* sp.) had the largest difference in frequency of occurrence between females and males (24.6% and 32.4%, respectively), while quaker moth (*Orthosia* sp.) was the invertebrate taxon which showed the largest frequency of occurrence difference between sexes (10.2% and 4.1% frequency of occurrence in females and males, respectively).

**TABLE 4:** Genus level frequency of occurrence and the difference between sexes (Δ).

Frequency of occurrence (%)				
Taxon	Plants/invertebrates	Female (*n* = 118)	Male (*n* = 145)	Δ
*Acer*	Plants	6.8	3.4	3.4
*Acleris*	Invertebrates	0	0.7	0.7
*Adela*	Invertebrates	0	0.7	0.7
*Aethalura*	Invertebrates	1.7	0	1.7
*Agelastica*	Invertebrates	0	2.1	2.1
*Agriopis*	Invertebrates	3.4	0	3.4
*Agrochola*	Invertebrates	2.5	0.7	1.8
*Aleimma*	Invertebrates	1.7	1.4	0.3
*Alnus*	Plants	1.7	1.4	0.3
*Amphipyra*	Invertebrates	8.5	6.2	2.3
*Andricus*	Invertebrates	0.8	0.7	0.1
*Anorthoa*	Invertebrates	0	1.4	1.4
*Anthoxanthum*	Invertebrates	0	1.4	1.4
*Anyphaena*	Invertebrates	2.5	2.8	0.3
*Apocheima*	Invertebrates	0.8	0	0.8
*Apotomis*	Invertebrates	0	0.7	0.7
*Araneus*	Invertebrates	0.8	0.7	0.1
*Archips*	Invertebrates	3.4	0	3.4
*Betula*	Plants	6.8	6.9	0.1
*Biorhiza*	Invertebrates	0	0.7	0.7
*Blastobasis*	Invertebrates	0.8	0	0.8
*Brassica*	Plants	0.8	0.7	0.1
*Carpinus*	Plants	16.1	14.5	1.6
*Charmon*	Invertebrates	0.8	0	0.8
*Cimbex*	Invertebrates	3.4	0.7	2.7
*Clausilia*	Invertebrates	0	0.7	0.7
*Clubiona*	Invertebrates	0	0.7	0.7
*Coleophora*	Invertebrates	0.8	0.7	0.1
*Colotois*	Invertebrates	0.8	4.1	3.3
*Conistra*	Invertebrates	7.6	6.2	1.4
*Cosmia*	Invertebrates	3.4	2.1	1.3
*Craniophora*	Invertebrates	0	0.7	0.7
*Culicoides*	Invertebrates	0.8	1.4	0.6
*Cyzenis*	Invertebrates	0.8	0	0.8
*Dactylis*	Plants	0	0.7	0.7
*Drepana*	Invertebrates	0.8	0.7	0.1
*Ectropis*	Invertebrates	2.5	0	2.5
*Egle*	Invertebrates	0	0.7	0.7
*Empoasca*	Invertebrates	0.8	0	0.8
*Epinotia*	Invertebrates	0	0.7	0.7
*Epirrita*	Invertebrates	7.6	6.2	1.4
*Epuraea*	Invertebrates	0	0.7	0.7
*Erannis*	Invertebrates	11.9	9	2.9
*Eriocrania*	Invertebrates	0.8	1.4	0.6
*Eudemis*	Invertebrates	5.1	0.7	4.4
*Eupithecia*	Invertebrates	1.7	0.7	1
*Eupsilia*	Invertebrates	7.6	5.5	2.1
*Fagus*	Plants	39	36.6	2.4
*Formica*	Invertebrates	0.8	0	0.8
*Fraxinus*	Plants	1.7	2.8	1.1
*Gypsonoma*	Invertebrates	1.7	1.4	0.3
*Hedera*	Plants	3.4	0	3.4
*Helianthus*	Plants	24.6	32.4	7.8
*Hemerobius*	Invertebrates	0	1.4	1.4
*Holcus*	Plants	0.8	0.7	0.1
*Hydriomena*	Invertebrates	0	0.7	0.7
*Hyposoter*	Invertebrates	0.8	0	0.8
*Ilex*	Plants	1.7	8.3	6.6
*Larix*	Plants	1.7	0.7	1
*Lehmannia*	Invertebrates	9.3	11	1.7
*Limax*	Invertebrates	0.8	0	0.8
*Neomyia*	Invertebrates	0.8	0	0.8
*Neriene*	Invertebrates	0	0.7	0.7
*Neuroterus*	Invertebrates	2.5	6.2	3.7
*Nothofagus*	Plants	1.7	1.4	0.3
*Oncopsis*	Invertebrates	0	0.7	0.7
*Operophtera*	Invertebrates	14.4	11.7	2.7
*Orthosia*	Invertebrates	10.2	4.1	6.1
*Pandemis*	Invertebrates	3.4	1.4	2
*Philodromus*	Invertebrates	0.8	1.4	0.6
*Phycita*	Invertebrates	1.7	0	1.7
*Phyllobius*	Invertebrates	0	0.7	0.7
*Poa*	Plants	0	2.1	2.1
*Polydrusus*	Invertebrates	0	0.7	0.7
*Prunus*	Plants	11	10.3	0.7
*Psoricoptera*	Invertebrates	0	0.7	0.7
*Ptycholoma*	Invertebrates	4.2	2.8	1.4
*Quercus*	Plants	21.2	15.9	5.3
*Quercusia*	Invertebrates	0	2.1	2.1
*Ranunculus*	Plants	0.8	0	0.8
*Rhynchaenus*	Invertebrates	1.7	2.1	0.4
*Rosa*	Plants	0.8	0.7	0.1
*Rubus*	Plants	0.8	2.1	1.3
*Salix*	Plants	0.8	1.4	0.6
*Satyrium*	Invertebrates	0	1.4	1.4
*Scathophaga*	Invertebrates	0.8	0	0.8
*Selenia*	Invertebrates	0.8	0	0.8
*Sorbus*	Plants	1.7	1.4	0.3
*Stomoxys*	Invertebrates	0.8	0	0.8
*Syrphus*	Invertebrates	0.8	0.7	0.1
*Taeniothrips*	Invertebrates	6.8	4.1	2.7
*Taraxacum*	Plants	0.8	0	0.8
*Taxus*	Plants	1.7	2.8	1.1
*Tetragnatha*	Invertebrates	0	0.7	0.7
*Thrips*	Invertebrates	1.7	5.5	3.8
*Tipula*	Invertebrates	0	0.7	0.7
*Tortricodes*	Invertebrates	5.1	6.2	1.1
*Ulmus*	Plants	6.8	7.6	0.8
*Urtica*	Plants	0	0.7	0.7
*Veronica*	Plants	0.8	0	0.8
*Viola*	Plants	0	0.7	0.7
*Vitrina*	Invertebrates	0	0.7	0.7
*Ypsolopha*	Invertebrates	0.8	0.7	0.1

## 4 DISCUSSION

Detailed dietary information is vital for understanding species' ecology and implementing effective conservation management plans (Chua et al., 2021). This study used a metabarcoding approach to provide the first comprehensive analysis of UK Hawfinch diet, as well as highlighting dietary differences between Hawfinch populations across the UK and between sexes. Furthermore, as found in other studies of bird diet (Davies et al., 2022; Jedlicka et al., 2017; McClenaghan et al., 2019; Sullins et al., 2018), a metabarcoding approach provided previously unknown and relevant data on Hawfinch diet composition not previously recorded using non‐molecular methods. Although this study documents over 100 taxa consumed, it also confirms that previously recorded common food resources such as beech, cherry, and Lepidoptera (Mountford, 1957; Newton, 1967) are frequently consumed. This highlights the power of metabarcoding techniques within bird ecology and in particular its relevance to passerines that feed on diverse and difficult to identify taxonomic groups, such as insects (da Silva et al., 2020; Davies et al., 2022).

Our results indicate that Hawfinch were consuming a wide range of dietary taxa. While dietary specialists may face greater constraints when responding to fluctuations in resource availability than generalist species, foraging can be a flexible activity (Tournayre et al., 2021; Twining et al., 2019). Our sampling provided 95.5% coverage of the possible dietary community Hawfinches consumed within their environment. The diversity estimates suggest that many taxa present in Hawfinch diet occurred rarely, as has been documented in previous fecal metabarcoding studies on generalist passerines (Shutt et al., 2020; Sottas et al., 2020). Many dietary taxa were consumed in very low frequencies by Hawfinch, suggesting they may be opportunistically consuming many species, but show consistent feeding on a small number of key dietary taxa. Our findings on the more frequent components of Hawfinch diet agree closely with previous observations of this species (Mountford, 1957). Previous studies found seeds of hornbeam, cherry, and maple were important throughout the year (Mountford, 1957). Our results, therefore, suggest evidence of a core diet of frequently consumed taxa and a secondary diet consisting of numerous rare taxa consumed infrequently. Hence, it could be concluded that Hawfinch may not be at risk from impacts of environmental changes affecting its dietary taxa distribution and abundance (Boyles & Storm, 2007; Fuller et al., 2005; Kirby et al., 2018; Twining et al., 2019). However, our results have revealed the presence of a core diet, which may be vital in maintaining Hawfinch fitness and population persistence. The taxa within this core diet may be threatened by local level landscape modification and, on a larger spatial scale, changes in woodland management. Future work, therefore, should evaluate the effects of the core and secondary dietary taxa variations on Hawfinch fitness, with specific focus on whether these variations may significantly impact demography and persistence of Hawfinch populations (Schweiger et al., 2015).

Buds of ash, maple, and beech, as well as Lepidoptera, became important food resources during spring and summer (Mountford, 1957). The importance of beech as a food resource was confirmed in this study, being the most prevalent plant taxon (detected in 38.5% of samples). It is well understood that birds must balance food handling times with net energy intake, and a resource is deemed more profitable if it has a higher energy reward per unit handling time (Molokwu et al., 2011). It is known that Hawfinch commonly feed on beech nuts during autumn and winter months (Mountford, 1957) due to the high fat and carbohydrate levels compensating for energy losses during winter (Renner et al., 2013). The onset of the breeding season can drive changes in feeding preferences as nutritional needs diversify (Lima, 2009). As the sampling in this study began during the pre‐breeding season and continued to the end of summer, Hawfinch may have been gaining a high energy reward from feeding on any remaining available beech nuts, but also obtaining similar nutritional benefits from the increased availability of beech buds in the spring. Beech buds have been shown to contain >15% fat (Lebl et al., 2010), and this may be an important energetic requirement for Hawfinch to boost condition before and during the breeding season.

Our results also revealed that Hawfinch consumed, albeit in low frequencies, herbaceous plant taxa such as dandelions (*Taraxacum*) and *Rubus* sp. This would indicate that Hawfinch are consuming these taxa while foraging on the ground or in the shrub layer during the breeding season. Hawfinch were previously thought to be purely canopy feeders during the breeding season (Perea et al., 2014); however, our results suggest that Hawfinch may be able to exploit seasonal food resources which occur in the lower canopy and on the ground.

Hawfinch egg laying begins around mid‐April (Kirby et al., 2019), and the presence of invertebrates within the diet during the breeding season is similar to other omnivorous passerine dietary studies conducted over similar temporal periods (Shutt et al., 2020). As invertebrate taxa were found more frequently in female diet than male, it can be presumed that invertebrates may help to provide specific nutrients beneficial to breeding physiology, such as egg production, as well as providing high protein food for chicks (Marshall et al., 2016). The dietary patterns found herein are commonly observed in other passerine species such as Chaffinch (*Fringilla coelebs*, Linnaeus; Holland et al., 2006). Lepidoptera, Coleoptera, Hemiptera, Annelida, Gastropoda, and Araneae were all observed as prey at the order level (Mountford, 1957), and all (excluding Annelida) were detected within this study. The high prevalence of winter moth within Hawfinch diet is not unexpected, as this larva is an important food resource for other woodland passerine species, such as nestling tits (Perrins, 1991). In contrast, the high prevalence of tree slug within the diet was unexpected, as it was previously thought only snails were consumed (Mountford, 1957). This may be explained by the availability of algae and lichens within woodland, which are the main components of tree slug diet (Kappes, 2006). During wet weather, tree slugs feed on algae growing on tree trunks, but remain under the bark of dead timber during unsuitable weather (Kappes, 2006). Thus, tree slugs may be taken during periods of high rainfall when foraging efficiency for defoliating Lepidoptera is reduced (Morganti et al., 2017; Ortega‐Jimenez & Dudley, 2012).

Spatial dietary differences observed between Hawfinch populations are consistent with similar metabarcoding studies of birds and insectivorous bats (Clare et al., 2014; Czenze et al., 2018; Shutt et al., 2020; Tournayre et al., 2021). This could indicate local dietary specialization; however, it is more probable that Hawfinch exhibit dietary plasticity and individual preference, with these patterns arising from changing availability of food resources between locations (Chua et al., 2021). The Wye Valley and north Cardiff regions occur within heterogeneous woodlands consisting of predominately beech and elm, while the north Wales region consisted of woodland supporting hornbeam and cherry. This differentiation in resource use is evident from the NMDS plot (Figure 3). While there is a degree of overlap between all regions, the Wye Valley and north Cardiff sampling regions are situated closer together, indicating dietary taxa detected from Hawfinch sampled within these regions show higher levels of similarity than dietary taxa from Hawfinch sampled in north Wales.

We revealed sexual differences in Hawfinch diet, likely due to behavioral and nutritional differences between males and females. Behavioral differences between sexes may be a factor due to differences in reproductive roles (da Silva et al., 2020; Freeman, 2014). This may be a result of differing requirements for reproduction and growth during the breeding season, for example, egg‐laying, with females facing a trade‐off between self‐maintenance and reproduction (García‐Campa et al., 2020). Female Hawfinch may be restricted in foraging due to nesting activities, while the higher mobility of males may enable them to forage for more nutritious prey taxa that are less abundant in the immediate environment around the nest site (da Silva et al., 2020). Furthermore, dietary composition differences between sexes may be a result of increased intraspecific competition during the breeding period. Hawfinch are judged to have minimal sexual dimorphism; however, biometric measurements such as bill length/depth were not recorded for this study, and therefore, future work should incorporate this to improve understanding of possible intra‐specific variation. Understanding nutritional requirements between Hawfinch sexes would also be an important consideration in understanding the sexual differences in diet seen within this study.

An important aspect to consider within any DNA metabarcoding study is prey detection biases, which can impact the results and subsequent ecological interpretation of metabarcoding studies (Forsman et al., 2022). The choice of primers is considered to be one of, if not the most important steps for reducing biases (Hoenig et al., 2022). The choice of primers can impact amplification efficiency and taxonomic classification of subsequent amplicon sequences (Brandon‐Mong et al., 2015). As a result, primer choice could influence the understanding of prey composition within the diet and thus the interpretations of foraging ecology (Alberdi et al., 2018; Forsman et al., 2022). Primer biases are considered particularly problematic when undetected taxa are ones which contribute substantially to the foraging ecology of the study species (Forsman et al., 2022). Hawfinch have previously been observed to feed mainly on Lepidoptera (Mountford, 1957), which was well represented at high frequency of occurrences across sites. The primer pair used in this study were originally used to characterize the diet of blackbirds (*Turdus merula*) and song thrushes (*Turdus philomelos*) and subsequently were designed to amplify a broad range of invertebrate taxa (Stockdale, 2018). It is important to note however that no primer pair can provide a completely unbiased and comprehensive account of species' diet due to highly degraded DNA failing to amplify in PCRs, primer biases and differences in mitochondrial copy number per cell (reviewed in Clare, 2014). A one‐locus‐several‐primer approach should be used more readily within DNA metabarcoding studies in order to maximize taxonomic coverage and minimize false negatives (Corse et al., 2019).

It is also important to acknowledge the possibility of secondary consumption via lepidopteran taxa within the diet, which may result in indirect species associations (Tercel et al., 2021). Secondary consumption can result in falsely inflated detection of plant taxa through co‐amplification of plant DNA within the guts of lepidopteran taxa consumed by Hawfinch. Ecologically, it is known that Hawfinch feed primarily within the canopy (Mountford, 1957; Perea et al., 2014). This suggests that most invertebrate taxa were obtained from the vegetation or bark within the tree canopy, resulting in possible accidental ingestion of plant taxa when gleaning prey items from trees. Due to metabarcoding methods being unable to determine which plant tissue is being consumed, in conjunction with Hawfinch also feeding on the same plant taxa as their prey at similar times of the year, differentiating what is “true” secondary predation in this study was extremely challenging.

It is important to note that these conclusions are based upon a small number of samples collected from north Cardiff (*n* = 7) and East Anglia (*n* = 7), such that inferences about UK wide variation in diet maybe somewhat speculative. To increase the spatial coverage shown within this study, future work should incorporate an increased number of samples collected across each sampling site, so that Hawfinch populations are better represented across them. Hawfinch are known to ground feed on fallen seed during winter months (Mountford, 1957); however, the winter diet of Hawfinch has not been explored in detail. This study has focused on Hawfinch diet between March–July, therefore, to improve knowledge of Hawfinch diet throughout the year, a wider sampling season should be undertaken. This will generate an insight in order to establish whether the “hunger gap” can be identified as a factor in Hawfinch decline, as identified in other seed‐eating species (Siriwardena et al., 2008). The lower number of samples testing positive for invertebrate DNA within the diet may be a result of the time of sampling. A significant number of fecal samples were collected between March and mid‐April (*n* = 138) when seasonal invertebrate activity is lower (Driessen et al., 2013).

In conclusion, this study has provided the first molecular insight into the generalist diet of Hawfinch, at a finer resolution than previous work. We demonstrate that the diet of Hawfinch, as predicted, varies both spatially and between sexes. This dietary variation suggests Hawfinch can respond to changing resource availability by showing dietary plasticity. The results of this study were only possible due to the high taxonomic resolution available through metabarcoding methods. As metabarcoding is becoming more prevalent within ecological research, it becomes increasingly important to understand how taxonomic resolution can impact ecological studies, although species‐level identification may not always be necessary, depending on hypotheses studied (Brown et al., 2014; Renaud et al., 2020). Finally, this study demonstrates how the utilization of DNA metabarcoding can increase ecological understanding and improve insights into fine scale ecological patterns.

## AUTHOR CONTRIBUTIONS

**Ewan H. Stenhouse:** Formal analysis (lead); writing – original draft (lead); writing – review and editing (lead). **Paul Bellamy:** Conceptualization (equal); funding acquisition (equal); investigation (equal); methodology (equal); supervision (lead); writing – original draft (equal); writing – review and editing (equal). **Will Kirby:** Methodology (equal); resources (lead); supervision (supporting); writing – original draft (supporting); writing – review and editing (supporting). **Ian P. Vaughan:** Formal analysis (equal); funding acquisition (equal); methodology (equal); software (equal); supervision (equal); writing – review and editing (equal). **Lorna E. Drake:** Resources (supporting). **Angela Marchbank:** Investigation (supporting); methodology (equal); resources (lead); supervision (supporting). **Trudy Workman:** Methodology (supporting); resources (supporting); supervision (supporting). **William O. C. Symondson:** Conceptualization (equal); funding acquisition (equal); supervision (lead). **Pablo Orozco‐terWengel:** Conceptualization (lead); funding acquisition (equal); supervision (equal); writing – review and editing (equal).

## CONFLICT OF INTEREST STATEMENT

The authors declare no conflict of interest.

### OPEN RESEARCH BADGES

This article has earned an Open Data badge for making publicly available the digitally‐shareable data necessary to reproduce the reported results. The data is available at https://doi.org/10.5061/dryad.0p2ngf25d.

## Supporting information


Appendix S1.

Appendix S2.

Appendix S3.

Appendix S4.

Appendix S5.

Data S1.

## Data Availability

All data supporting the work presented in this study are openly available on Dryad at https://doi.org/10.5061/dryad.0p2ngf25d.
