# Supplementary material for: Multi‐marker DNA metabarcoding reveals spatial and sexual variation in the diet of a scarce woodland bird
Source: Ecol Evol. 2023 May 17;13(5):e10089. doi: 10.1002/ece3.10089 (PMC10191781; doi:10.1002/ece3.10089)
Supplement: Supplementary file 6 — Data S1. [file ECE3-13-e10089-s006.docx]

**Appendix Metadata**

Excel files showing a cleaning pipeline for raw Illumina sequences post bioinformatic analysis. Cleaning pipelines are separated by individual libraries included in the Illumina sequencing run.

**Column headers**

For cleaning - Raw Illumina sequencing reads

Contam removed - reads remaining in samples following the removal of maximum number of contamination reads

FOO per sample – The Frequency of occurrence per reads in that sample. Calculated by dividing the number of reads of that taxon in that sample by the total number of reads found for all taxa in the same sample

% per sample – FOO per sample *100

> 1% occurrence per sample – Taxa are highlighted if FOO of reads of a taxon make up more than 1% of total number of reads in that sample

> 2% occurrence per sample – Taxa are highlighted if FOO of reads of a taxon make up more than 2% of total number of reads in that sample

> 3% occurrence per sample – Taxa are highlighted if FOO of reads of a taxon make up more than 3% of total number of reads in that sample

> 4 occurrence per sample – Taxa are highlighted if FOO of reads of a taxon make up more than 4% of total number of reads in that sample

>5 occurrence per sample – Taxa are highlighted if FOO of reads of a taxon make up more than 5% of total number of reads in that sample

>8 occurrence per sample Taxa are highlighted if FOO of reads of a taxon make up more than 8% of total number of reads in that sample

Keep >1% sample filtering – Only retain sequencing reads for taxa if % per sample is more than 1% of total number of reads in that sample

Keep >2% sample filtering – Only retain sequencing reads for taxa if % per sample is more than 2% of total number of reads in that sample

Keep >3% sample filtering – Only retain sequencing reads for taxa if % per sample is more than 3% of total number of reads in that sample

Keep >4% sample filtering – Only retain sequencing reads for taxa if % per sample is more than 4% of total number of reads in that sample

Keep >5% sample filtering – Only retain sequencing reads for taxa if % per sample is more than 5% of total number of reads in that sample

Keep >8% sample filtering – Only retain sequencing reads for taxa if % per sample is more than 8% of total number of reads in that sample

Keep >15% sample filtering – Only retain sequencing reads for taxa if % per sample is more than 15% of total number of reads in that sample

Max contam + 3% filter – Only keep reads for taxa if reads are still present after contamination removal and the % of reads per sample is more than 3% of total number of reads in that sample

Max contam + 5% filter – Only keep reads for taxa if reads are still present after contamination removal and the % of reads per sample is more than 5% of total number of reads in that sample

Max contam + 8% filter – Only keep reads for taxa if reads are still present after contamination removal and the % of reads per sample is more than 8% of total number of reads in that sample
